# A longitudinal molecular surveillance of genetic heterogeneity of *Orientia tsutsugamushi* in humans, reservoir animals, and vectors in Puducherry, India

**DOI:** 10.3389/fmicb.2025.1634394

**Published:** 2025-08-29

**Authors:** Krishan Kumar Sihag, Waseema Arif, Srikanth Srirama, Anand Kumar Chandrasekaran, Vinod Raveendran, Asayas Bosco Chandrakumar, Anand Kasirajan, Sivagamy Alias Punitha Thavaraj, Lakshmy Srinivasan, Anoop C. Choolayil, Mathivanan Ashokkumar, Amala Ramasamy, Nanda Kumar Yellapu, Panneer Devaraju

**Affiliations:** ^1^Unit of One Health, ICMR-Vector Control Research Centre (ICMR-VCRC), Indira Nagar, Puducherry, India; ^2^ICMR-Vector Control Research Centre (ICMR-VCRC), Affiliated to Pondicherry University, Kalapet, Puducherry, India; ^3^Indira Gandhi Medical College and Research Institute, Kathirkamam, Puducherry, India; ^4^Sri Venkateshwaraa Medical College Hospital and Research Centre, Ariyur, Puducherry, India; ^5^Sri Lakshmi Narayana Institute of Medical Science, Villianur, Puducherry, India; ^6^Mahatma Gandhi Medical Advance Research Institute, Sri Balaji Vidyapeeth, Pilayarkuppam, Puducherry, India; ^7^ICMR-Vector Control Research Centre (ICMR-VCRC), Indira Nagar, Puducherry, India; ^8^Unit of Microbiology and Immunology, ICMR-Vector Control Research Centre (ICMR-VCRC), Indira Nagar, Puducherry, India; ^9^Unit of Biostatistics and VBD Modeling, ICMR-Vector Control Research Centre (ICMR-VCRC), Indira Nagar, Puducherry, India; ^10^Unit of Clinical and Molecular Medicine, ICMR-Vector Control Research Centre (ICMR-VCRC), Indira Nagar, Puducherry, India

**Keywords:** scrub typhus, molecular diagnostics, genetic heterogeneity, TA678-like strain, phylogenetic analysis

## Abstract

**Background:**

Scrub typhus (ST), is a vector borne zoonotic disease, transmitted by the larva of the trombiculid mites. The enzootic cycle of the pathogen involves rodents/shrews as the animal reservoirs and humans are the accidental dead-end host. A transposon-like activity in its major antigen 56 kDa, has led to the evolution of several serotypes/strains, and more than 40 serotypes are reported globally. Puducherry, India, is endemic to scrub, but limited data exist on local serotype distribution across hosts and vectors.

**Methodology:**

A longitudinal molecular surveillance was conducted in Puducherry to investigate the genetic diversity of *Orientia tsutsugamushi* among humans, animal reservoirs, and vectors. Samples from febrile patients, trapped rodents/shrews, and their infesting mites were screened using real-time and nested PCR. Serotype analysis was performed by partial amplification and sequencing of the 56 kDa gene, followed by phylogenetic, pairwise genetic distance and amino acid analysis.

**Result:**

ST infection was detected in 4.37% (95% CI: 3.05–5.71%) of human, 11.52% (95% CI: 8.6–14.4%) of rodent/shrew, and 2.36% (95% CI: 0.95–4.87%) of mite samples. Karp-like (51.72%) and Gilliam-like (41.38%) strains were predominant in both humans and animal hosts, with pairwise genetic distance (<0.1) and amino acid identity (>85%) analysis revealing a close relationship between the strains identified across the region. Notably, the only mite pool that tested positive for the 56 kDa gene, along with a shrew, was identified to belong to the TA678-like serotype (6.90%), which has not been previously reported from Puducherry.

**Conclusion:**

This study provides molecular evidence of the enzootic maintenance and active human transmission of *O. tsutsugamushi* in Puducherry, with multiple co-circulating serotypes. The first detection of the TA678-like strain in the region suggests the possible introduction of new strains and underscores the need to monitor for strain-specific clinical manifestations in future studies.

## 1 Introduction

Scrub typhus (ST) is a re-emerging rickettsial disease caused by *Orientia tsutsugamushi*, an obligate intracellular Gram-negative bacterium. It is transmitted by the bite of the chigger mites, majorly, by the trombiculid mites, e.g., *Leptotrombidium deliense*. In humans, the infection presents as an acute undifferentiated febrile illness (AUFI) with wide-ranging severity. The disease is associated with mortality rates of up to 70% (median rate 6%) in untreated cases, with over 1 billion humans at risk of acquiring the infection globally (Taylor et al., 2015). It is endemic in the Southeast Asian countries, constituting the tsutsugamushi triangle, and has recently exhibited its extension to other regions as well. Since the identification of three antigenically diverse strains of *O. tsutsugamushi* by Shishido, there have been reports of multiple strains that have caused several outbreaks (Shishido, 1962; Seetha et al., 2023). The 56 kDa gene encodes the most abundant antigen, the outer membrane protein of *O. tsutsugamushi*. The hypervariable regions in the 56 kDa gene contribute to the array of pathogenic strains (Ohashi et al., 1992). Based on the genetic diversity of the 56 kDa gene more than 40 serotypes have been reported, with their distribution changing from region to region in the tsutsugamushi triangle. The Karp and Gilliam serotypes are prevalent in Taiwan, Gilliam in China, and the Boryong in South Korea. In India Kato and Karp are dominant, while Kato, Karp, Gilliam, Kawasaki, and Kuroki types are found in Japan (Prakash, 2017). More importantly, the clinical characteristics of the disease were observed to vary with the serotype of the organism. The Karp strain is often linked to severe illness including encephalitis and multi-organ dysfunction, while strains like Boryong and Kato are generally associated with milder clinical presentations. Further, multiple ecological factors including the number of rodents, the characteristics of the habitat, the chigger index and the climatic conditions also play a critical role in the incidence of ST (Bhopdhornangkul et al., 2021; Ding et al., 2022; Chang et al., 2024; Konyak et al., 2024).

India has reported several outbreaks of ST with consistent disease burden in the past few decades. Puducherry is one among the endemic areas in the country reporting an increasing trend in the number of cases reported annually. The figures reached a maximum of 998 cases in 2022 and 984 cases in 2023. Subsequently, numerous studies were conducted to estimate the prevalence of ST in human and animal reservoirs. However, only a few have focused on the molecular characterization of the circulating serotypes of *O. tsutsugamushi* by amplification and nucleotide sequencing of the 56-kDa gene. Previous molecular studies in humans from Puducherry targeting the 56-kDa gene have reported a prevalence of 27.6 and 30% but did not determine the prevailing serotype (Patricia et al., 2017; Anitharaj et al., 2020). In the case of small mammals, Candasamy et al., did not observe any positivity for the 56-kDa gene in rodents/shrews collected from Puducherry (Candasamy et al., 2016). Recently, in household rats from Tamil Nadu, Karnataka, and Puducherry, Purushothaman et al., reported a prevalence of 55.29% by targeting the 47-kDa gene (Purushothaman et al., 2024). Notably, these studies too did not explore the serotype of the pathogen. In our previous works we observed the circulation of Karp, Kato and Kawasaki serotypes in the reservoirs and mite vectors in Puducherry (Devaraju et al., 2020; Balasubramanian et al., 2024; Eikenbary et al., 2024; Ritu et al., 2024). In continuation, the current study aimed to identify the serotype of the pathogen circulating among the patients (scrub typhus confirmed by PCR) and to establish its association with the strains circulating among the vector mites and the reservoir hosts rodents/shrews in Puducherry. Understanding the serotype distribution is crucial for refining the existing diagnostic assays, developing novel diagnostic tools, and advancing vaccine research (Koraluru et al., 2015).

## 2 Materials and methods

### 2.1 Ethical approval for study on rodent/shrews and human participants

The trapping and collection of blood and organ samples from the trapped rodents/shrews was approved by the Institutional Animal Ethics Committee (No: ICMR-VCRC/IAEC/2018/2) of ICMR-VCRC. For the use of human blood samples, the Institutional Human Ethics Committee (IHEC 1022/N/F) of ICMR-VCRC approval was obtained.

### 2.2 Human sample collection

All experiments on human participants were performed in accordance with the Declaration of Helsinki. Prior informed consent from human participants and/or their legal guardians were obtained before the sample collection. Blood samples were collected from patients (classified as acute undifferentiated febrile illness), with history of fever lasting between 3 to 14 days without any evident foci. The study excluded patients with severe illness precluding informed consent, those receiving immunosuppressive therapy, and individuals with a prior diagnosis of autoimmune disorders or malignancies. Additionally, patients presenting with an identifiable source of infection or an alternative cause of fever, as determined during the initial clinical evaluation by the attending physician, were also excluded. Two milliliters of blood in EDTA vials were collected from the patients seeking medical facilities from three tertiary care centers at Puducherry [1. Sri Venkateshwaraa Medical College Hospital and Research Centre (SVMCH&RC), 2. Sri Lakshmi Narayana Institute of Medical Science (SLIMS), and 3. Indira Gandhi Medical College and Research Institute (IGMCRI)] for 19 months from August 2022 to February 2024.

### 2.3 Trapping and processing of rodents/shrews

ARRIVE guidelines were followed with the animal usage in this study. Rodents/shrews were trapped from 29 randomly selected study sites in and around Puducherry. The live trapping of rodents/shrews was achieved using Sherman traps, using food items made of flour (“pakoda”) as bait. The rodents/shrews were trapped for a period of 1 year from January 2022 to December 2022. Traps (*n* = 20/site) were placed in domestic and peri-domestic areas around human habitats, vegetations and at the site of rodent/shrew burrows. The traps were set 1–2 h before the sunset and were retrieved before dawn. The positive traps were transported to the laboratory for further processing. The trapped rodents were euthanised with CO_2_ as recommended by CCSEA (Committee for the Purpose of Control and Supervision of Experiments on Animals (CPCSEA), 2018) and taxonomically identified based on the morphological features (Agrawal, 2000).

From the trapped rodents/shrews, blood was collected by cardiac puncture in vials containing EDTA. Additionally, organs including the lungs, heart, brain, spleen, kidneys, liver, and small intestine were dissected and transferred to a labeled container with sterile PBS and then stored at −40°C until DNA isolation.

### 2.4 Ectoparasite collection from the trapped rodents/shrews

The trapped rodents/shrews were examined for the mite infestation. Using tweezers and hair brush, the mites in the pinnae, legs and fur of rodents/shrews were retrieved and stored in 70% ethanol at room temperature. Taxonomical identification based on the morphological features was carried out randomly in 2% of the mites. The taxonomically identical mites were grouped into pools (1–40 mites/pool) according to the geographical site of trapping and host.

### 2.5 Processing of the samples

Extraction of DNA from human and rodent/shrew blood samples was done using the DNeasy Blood and Tissue Kit (Qiagen, Hilden, Germany) following the manufacturer’s instructions. DNA from the mites and rodent/shrew organ samples was extracted by phenol-chloroform method (Ritu et al., 2024). Briefly, organs and mite pools were mechanically lysed using metallic beads and a tissue lyser (Qiagen, Hilden, Germany), followed by enzymatic digestion with proteinase K and lysis buffer. DNA was extracted using phenol-chloroform-isoamyl alcohol and purified through ethanol precipitation. The final DNA pellet was air-dried and reconstituted in nuclease-free water for downstream applications.

### 2.6 Molecular detection of *O. tsutsugamushi*

Molecular screening of *O. tsutsugamushi* in the DNA extracted from AUFI patients, rodents/shrews and mite pools was carried out by following the published protocols using the Real-time PCR (Tantibhedhyangkul et al., 2017) and Nested PCR (Furuya et al., 1993). All oligonucleotide primers and probes used in this study were procured from Eurofins Genomics India Pvt. Ltd. (Bangalore, Karnataka). Real-time PCR targeting the 47 kDa gene was performed using primers FP: 5′-CCATCTAATACTGTACTTGAAGCAGTTGA-3′; RP: 5′-GTCCTAAATTCTCATTTAATTCTGGAGT-3′ and a TaqMan probe FAM-TCATTAAGC/ZEN/ATAACATTTAACATACCACGACGA-IBFQ. Amplification was carried out using the Roche LightCycler® 96 system in a 10 μL reaction volume containing 2X LightCycler® 480 Probes Master (Roche Diagnostics, Mannheim, Germany), 10 pmol/μL each of forward and reverse primers, 10 pmol/μL of TaqMan probe, and template DNA. Thermal cycling conditions were: 95°C for 5 min, followed by 45 cycles of 95°C for 10 s, 60°C for 20 s, 72°C for 1 s, and a final step at 37°C for 30 s. Samples with a Ct value <40 were considered positive.

Two-step nested PCR targeting the 56 kDa type-specific antigen (TSA) gene was conducted using oligonucleotide primers N1 FP: 5′-TCAAGCTTATTGCTAGTGCAATGTCTGC-3′, N1 RP: 5′-AGGGATCCCTGCTGCTGTGCTTGCTGCG-3′, N2 FP: 5′-GATCAAGCTTCCTCAGCCTACTATAATGCC-3′, N2 RP: 5′-CTAGGGATCCCGACAGATGCACTATTAGGC-3′ as per Furuya et al. (1993). The first-round PCR was performed in a 25 μL reaction mixture containing 2X GoTaq® Green Master Mix (Promega, Madison, WI, USA), 10 pmol/μL of each primer, 25 mM MgCl_2_, and template DNA. The resulting amplicon was diluted 1:50 and used as the template for the second-round PCR. Thermocycling conditions for the first round were: 94°C for 5 min; 31 cycles of 94°C for 50 s, 55°C for 2 min, and 72°C for 2 min; followed by a final extension at 72°C for 7 min. The second-round PCR was carried out under the following conditions: 94°C for 5 min; 35 cycles of 94°C for 50 s, 58.8°C for 2 min, and 72°C for 2 min; followed by a final extension at 72°C for 7 min. PCR products were resolved by agarose gel electrophoresis, and the presence of a 483 bp amplicon was considered indicative of a positive result for *O. tsutsugamushi.*

### 2.7 Nucleotide sequencing and phylogenetic analysis

The 483 bp amplicons of 56 kDa TSA gene obtained from the nested PCR positive samples were subjected to Sanger sequencing using the Applied Biosystems Genetic Analyzer 3130XL (USA). Briefly, the PCR amplicons were purified using the NucleoSpin® Gel and PCR Clean-up kit (Macherey-Nagel, Duren, Germany) and the cycle sequencing reaction was set up using the BigDyeTM Terminator v3.1 Cycle Sequencing kit (Applied Biosystems, USA). The thermocycling conditions for the cycle sequencing PCR were: 96°C for 1 min and 25 cycles of 96°C for 10 s, 50°C for 5 s, 60°C for 4 min. The product was further purified using the NucleoSeq® kit (Macherey-Nagel, Duren, Germany), and subjected to nucleotide sequencing using the Applied Biosystems Genetic Analyzer 3130XL (USA). The quality of the Sanger sequencing results was checked using Chromas and edited in BioEdit. Sequences with clear, sharp peaks and low background noise were selected. The low quality reads at the beginning and end of each sequence were trimmed, and only those with good quality (Phred score ≥20) were used for further analysis. These high-quality sequences were then compared with known sequences in the NCBI database using BLASTn. Only matches with 97% or higher identity were accepted for identifying the strain. Multiple sequence alignment was done using ClustalW, and the phylogenetic tree was constructed by the Maximum Likelihood method with 1,000 bootstrap replicates using the Kimura-2 parameter model in the MEGA version 11 software (Tamura et al., 2021). Additionally, a distance matrix was generated based on pairwise genetic distances between the human, animal and mite 56 kDa sequences. The time scale analysis following Bayesian phylogenetics was performed using the HKY substitution model with strict molecular clock and constant population size tree prior in the BEAST package v2.7.7. The Markov Chain Monte Carlo analyses was run with 10,00,000 steps, combined with 10% burn value and a maximum clade credibility tree was constructed with 95% highest posterior density (HPD) interval using the TreeAnnotator and visualized using Figtree v1.4.4. Further, multiple sequence alignment of the translated amino acid sequences with the full-length standard reference sequence was carried out for each serotype using BioEdit software version 7.7.1. The amino acid changes observed were presented as a table and the percentage identity was determined.

### 2.8 Quality control measures

DNA extraction, PCR setup, and amplification were carried out in physically separated, designated laboratory areas to minimize the risk of cross-contamination. Standard operating procedures were followed at each stage including the DNA extraction, amplification, post-amplification, and sequencing. The quality of extracted DNA was assessed by spectrophotometric analysis (A260/A280 ratio) using a Nanodrop instrument (Thermofisher, Madison, WI, USA). Each PCR run included internal positive and negative controls to monitor amplification performance. For sequencing, the post-amplification control supplied with the BigDye Terminator v3.1 Cycle Sequencing Kit (Applied Biosystems, USA) was used to confirm the reliability of reactions. All sequences generated were submitted to NCBI GenBank.

### 2.9 Statistical analysis

The data was entered and maintained in Microsoft Excel and analysis was performed in STATA version 18 (StataCorp, 2023). Prevalence of infection among the humans, rodents/shrews and mites were expressed as percentage with 95% Confidence interval.

## 3 Results

### 3.1 Molecular prevalence of *O. tsutsugamushi* in humans

A total of 916 blood samples from patients with AUFI were collected from three collaborating tertiary care centers in Puducherry between August 2022 and February 2024. Among these, 39 samples tested positive for the 47 kDa HtrA gene by real-time PCR, and 17 samples were positive for the 56 kDa TSA gene by nested PCR (Supplementary Figure S1). Overall, 40 patients tested positive for scrub typhus (ST) by either the 56 kDa or 47 kDa PCR assays (4.37%; 95% CI: 3.05–5.71%), and 16 patients were positive by both tests (Supplementary Table S1). The ST-positive patients (*n* = 40) were aged between 18 and 75 years, comprising 24 males (60%) and 15 females (37.5%); data for one patient were not available. The majority of cases (*n* = 24, 60%) were in the age group of 30–60 years.

### 3.2 Molecular prevalence of *O. tsutsugamushi* in rodents/shrews

A total of 460 rodents/shrews were captured using Sherman traps deployed across 29 study sites over the course of 1 year, from January to December 2022 of which 261 (56.73%) were male and 199 (43.26%) were female. Month-wise trap success rate ranged between 10 and 28.18% (Supplementary Table S2) whereas, area-wise trap success rate was within a range of 5–75% (Supplementary Table S3). Overall, the average trap success rate was 20.18%. Among the rodents trapped, the majority were *Suncus murinus* (*n* = 373, 81.08%), followed by *Rattus rattus* (*n* = 72, 15.65%) and *Bandicota indica* (*n* = 15, 3.26%) (Table 1). In the trapped animals, mite infestation was found in 39.78% (*n* = 183). For molecular detection of ST in trapped rodents/shrews, along with blood, seven different organs such as heart, brain, lungs, small intestine, spleen, kidney and liver were screened. In total, 58 samples—including 19 blood, 21 lungs, 5 heart, 7 liver, 2 kidney, 3 brain and 1 intestinal sample—from 45 rodents/shrews (9.78%; 95% CI: 7.07–12.5%) were tested positive for 47 kDa HtrA gene by real-time PCR. Further, 24 samples—including 9 blood, 5 lungs, 3 heart, 2 liver, 1 kidney, 2 spleen and 2 intestinal samples—from 20 rodents/shrews (4.35%; 95% CI: 2.48–6.21%) were tested positive for 56 kDa gene by nested PCR (Table 1 and Supplementary Table S4). Overall, 53 (11.52%; 95% CI: 8.6–14.4%) animals including 4 *Rattus rattus* and 49 *Suncus murinus* were identified to harbor the bacteria in blood or any other tissue either by 47 kDa or by 56 kDa PCR. In addition, 12 animals were positive for both the tests in at least one of the tissues analyzed (Supplementary Table S4).

**Table 1:** Molecular detection of *Orientia tsutsugamushi* among infected humans, rodents/shrews, and chigger mites in and around Puducherry.

Sl. No.	Source of isolates	Total number of isolates/pools tested	Real time PCR positives	Nested PCR positives
1	Human			
	Whole blood	916	39	17
2	Rodents/shrews			
	*Suncus murinus*	373	42	18
	*Rattus rattus*	72	3	2
	*Bandicota indica*	15	0	0
3	Mite pools	296	7	1

### 3.3 Molecular prevalence of *O. tsutsugamushi* in chigger mites

**Figure 1:**
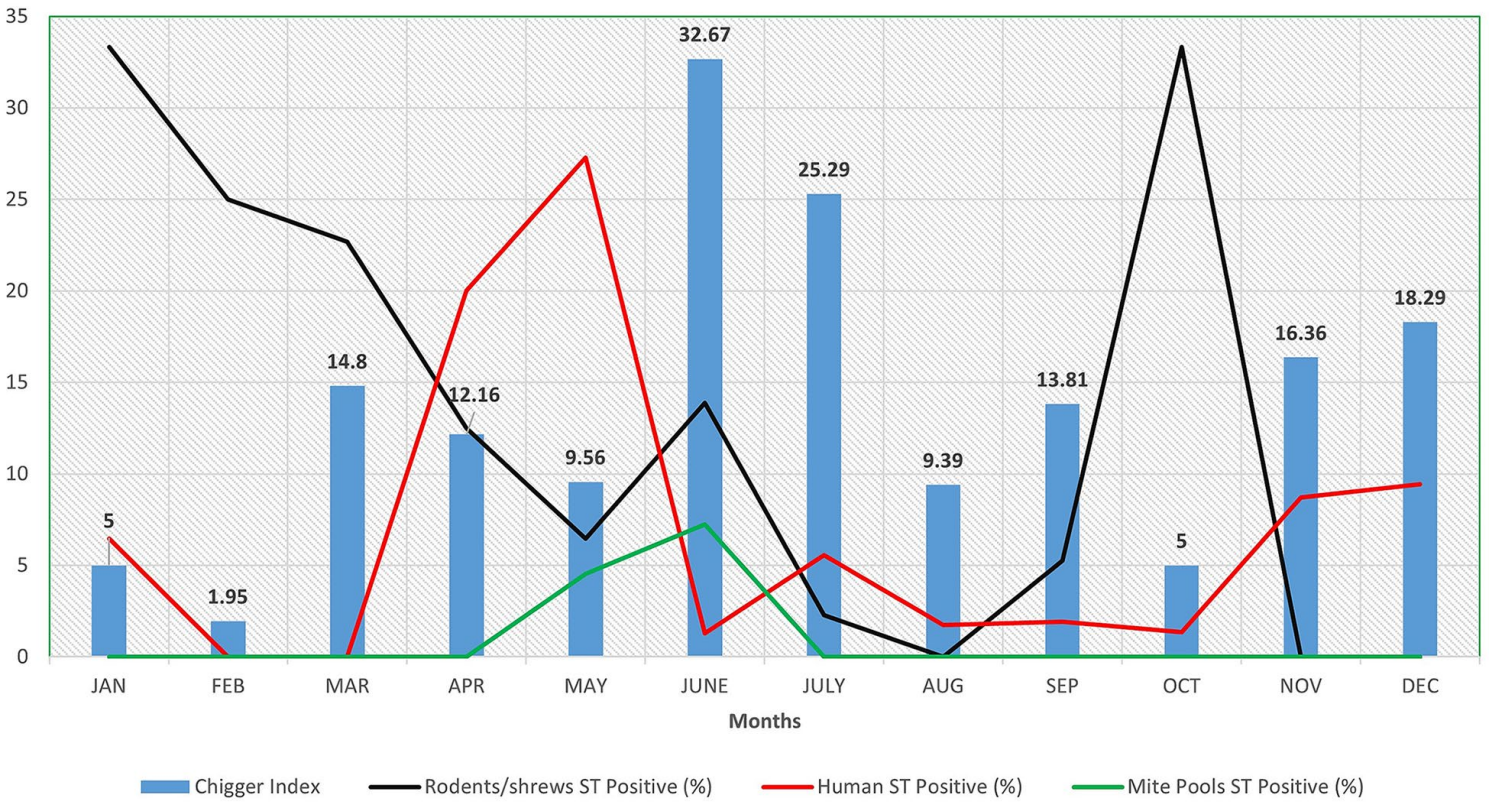
Graph depicting month wise chigger index along with scrub typhus positive (%) in rodents/shrews, humans and mite pools in and around Puducherry.

From the animals trapped, a total of 7,465 trombiculid mites were retrieved. Of the mites identified using morphological features, the majority of them belonged to the genus *Leptotrombidium* (78.87%), including *L. insigne* (50.70%) and *L. deliense* (28.17%). The chigger index per animal was 16.23, with the highest value of 32.67 recorded in the month of June (Figure 1). Among the 296 mite pools screened for the presence of ST, 7 (2.36%; 95% CI: 0.95–4.87%) mite pools from 5 shrews were positive for ST by real-time PCR. Only one pool was tested positive for 56 kDa gene by nested PCR and was also identified to be the only sample positive for both tests (Table 1).

### 3.4 Phylogenetic analysis of *O. tsutsugamushi* strains circulating in Puducherry

**Figure 2:**
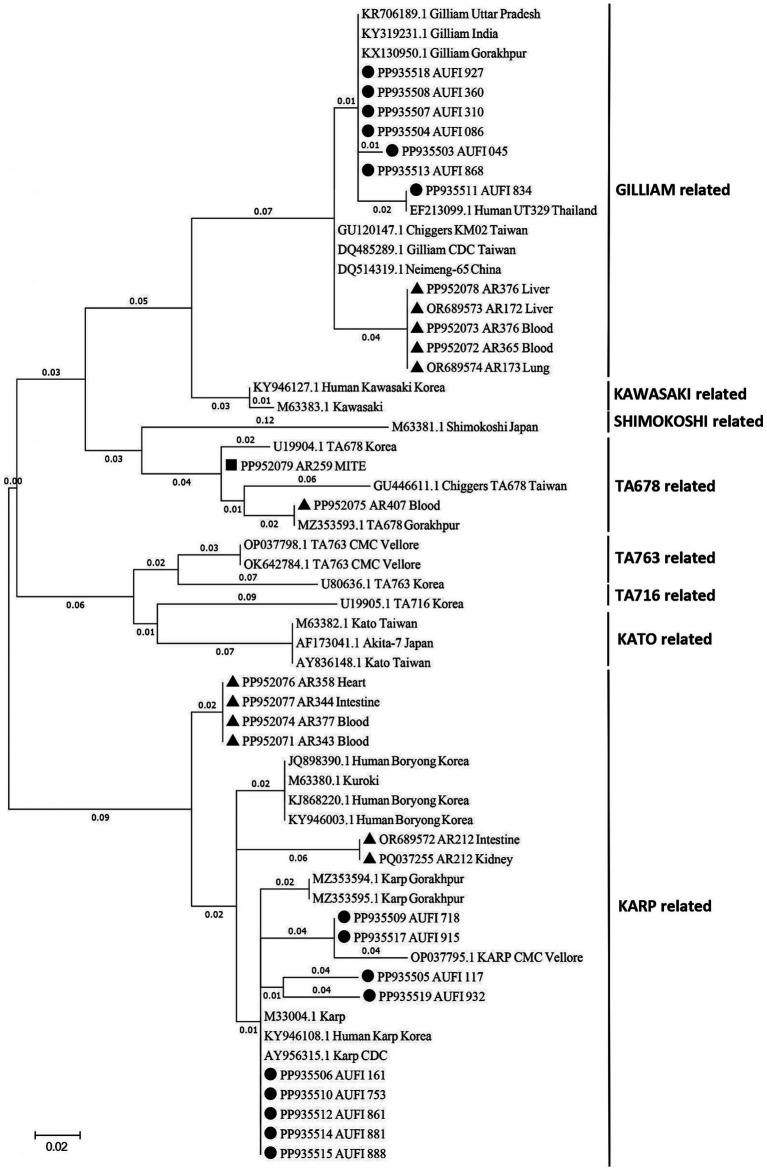
Phylogenetic analysis of *Orientia tsutsugamushi* sequences derived from animal reservoirs, humans, and chigger mites. The tree was constructed using the Maximum Likelihood method with 1,000 bootstrap replicates based on the Kimura 2-parameter model, targeting the 56-kDa type-specific antigen gene. In the current study, strains identified from rodent/shrew samples are denoted by TRIANGLES, those from human samples by CIRCLES, and those from mite samples by SQUARES.

Out of 42 nested PCR positive samples from humans (*n* = 17), rodents/shrews (*n* = 24) and vectors (*n* = 1), a total of 29 samples (16 human samples, 12 rodent/shrew samples, and 1 mite pool sample) yielded good quality nucleotide sequences which were subjected to genetic characterization. The sequences were analyzed and deposited in NCBI GenBank^1^ with the accession numbers listed in Supplementary Table S5. In the phylogenetic analysis (Figure 2) of the human samples (*n* = 16), the predominance of Karp-like (*n* = 9; 56.25%) and Gilliam-like (*n* = 7; 43.75%) strains was observed. Similarly, the dominant serotypes observed to be enzooticaly circulated among the animal reservoirs were identified to be Karp-like (*n* = 6; 50.00%) followed by Gilliam-like (*n* = 5; 41.67%) and TA678-like (*n* = 1; 8.33%). The only mite pool that was positive for 56 kDa was identified as TA678-like serotype. Interestingly, we observed that the animal host from which the only positive mite pool was retrieved, was tested negative for ST by both the real time and nested PCR assays.

From the phylogenetic tree constructed using MEGA v. 11.0., it is notable that the strains of *O. tsutsugamushi* identified in patients, rodents/shrews and mite pools clustered into 3 groups. The pairwise genetic distance between humans and rodents/shrews/mite samples varied widely ranging from 0.03 to 0.93. Majority of the bacterial strains identified in the humans exhibited a closer genetic relatedness (distance < 0.1) with one or more of the strains circulating in rodents/shrews. The Gilliam related bacterial strain identified in a patient, with the sequence ID: PP935511, was identified to exhibit the least genetic distance (0.03) from five other strains identified in rodents/shrews (PP952072, PP952073, PP952078, OR689573 and OR689574) (Table 2). The tree constructed following the Bayesian phylogenetic exhibited the similar topology as the maximum likelihood tree. The mean evolutionary rate was determined to be 3.94 × 10^−3^ mutations/site/year (95% HPD: 3.15 × 10^−3^–4.73 × 10^−3^) and the time to the most recent common ancestor (TMRCA) was estimated to be 167 years (95% HPD: 134.61–200.63 years) (Supplementary Figure S2).

**Table 2:** Distance matrix depicting the genetic distance between the sequences of 56 kDa antigen of *Orientia tsutsugamushi* isolated from humans, rodents, and mites in and around Puducherry.

Human samples	Animal samples												Mite
	PP952071	PP952072	PP952073	PP952074	PP952075	PP952076	PP952077	PP952078	OR689572	PQ037255	OR689573	OR689574	PP952079
PP935503	0.28	0.12	0.12	0.28	0.33	0.28	0.28	**0.09** ^*^	0.24	0.38	0.12	0.12	0.31
PP935504	0.23	**0.09** ^*^	**0.09** ^*^	0.23	0.45	0.24	0.25	**0.10** ^*^	0.24	0.33	**0.08** ^*^	**0.08** ^*^	0.33
PP935505	0.50	0.59	0.61	0.50	0.72	0.53	0.54	0.41	0.55	0.93	0.62	0.63	0.54
PP935506	**0.05** ^*^	0.30	0.30	**0.05** ^*^	0.46	**0.06** ^*^	**0.06** ^*^	0.27	**0.06** ^*^	**0.08** ^*^	0.29	0.30	0.32
PP935507	0.23	**0.08** ^*^	**0.07** ^*^	0.23	0.45	0.23	0.24	**0.09** ^*^	0.22	0.27	**0.07** ^*^	**0.07** ^*^	0.31
PP935508	0.23	**0.07** ^*^	**0.07** ^*^	0.23	0.42	0.23	0.24	**0.08** ^*^	0.24	0.34	**0.08** ^*^	**0.08** ^*^	0.31
PP935509	**0.08** ^*^	0.29	0.28	**0.08** ^*^	0.41	**0.08** ^*^	**0.08** ^*^	0.26	**0.05** ^*^	**0.06** ^*^	0.29	0.30	0.32
PP935510	**0.05** ^*^	0.30	0.30	**0.05** ^*^	0.41	**0.06** ^*^	**0.06** ^*^	0.28	**0.06** ^*^	**0.08** ^*^	0.30	0.31	0.32
PP935511	0.27	**0.03** ^*^	**0.03** ^*^	0.27	0.41	0.27	0.28	**0.03** ^*^	0.27	0.33	**0.03** ^*^	**0.03** ^*^	0.32
PP935512	**0.05** ^*^	0.30	0.30	**0.05** ^*^	0.40	**0.06** ^*^	**0.06** ^*^	0.28	**0.06** ^*^	**0.08** ^*^	0.30	0.31	0.32
PP935513	0.28	**0.06** ^*^	**0.06** ^*^	0.28	0.37	0.29	0.30	**0.03** ^*^	0.28	0.37	**0.06** ^*^	**0.06** ^*^	0.33
PP935514	**0.05** ^*^	0.30	0.30	**0.05** ^*^	0.44	**0.06** ^*^	**0.06** ^*^	0.28	**0.06** ^*^	**0.07** ^*^	0.30	0.31	0.32
PP935515	**0.05** ^*^	0.30	0.30	**0.05** ^*^	0.44	**0.06** ^*^	**0.06** ^*^	0.28	**0.06** ^*^	**0.07** ^*^	0.30	0.31	0.32
PP935517	**0.08** ^*^	0.29	0.28	**0.08** ^*^	0.43	**0.08** ^*^	**0.08** ^*^	0.26	**0.05** ^*^	**0.06** ^*^	0.28	0.29	0.32
PP935518	0.23	**0.07** ^*^	**0.07** ^*^	0.23	0.42	0.23	0.24	**0.08** ^*^	0.24	0.34	**0.08** ^*^	**0.08** ^*^	0.31
PP935519	**0.08** ^*^	0.19	0.18	**0.08** ^*^	0.25	**0.08** ^*^	**0.08** ^*^	0.18	**0.07** ^*^	0.13	0.18	0.19	0.23

Values which are highlighted using bold font and an “*” indicate close association of the pathogenic strains isolated from human and animal samples..

A table describing the amino acid changes in each strain in comparison with the reference was constructed and included in Supplementary Table S6. Among the Gilliam-type sequences, alignment with the reference strain (accession no. DQ485289.1) revealed that the sequence PP935507 exhibited the highest amino acid identity (94.16%), while the sequence PP952078 demonstrated the lowest identity (85.58%). For the Karp-type sequences, comparison with the reference strain (accession no. AY956315.1) revealed that the sequences PP935514, and PP935515 exhibited the highest identity (98.01%) while the sequence PP935505 exhibited the lowest identity (53.13%). Similarly, the TA678-like study sequences PP952075 and PP952079 exhibited 81.88 and 95.10% identity, respectively, when aligned with the reference strain (accession no. GU446611.1). As TA678 was reported for the first time in Puducherry it was also compared with the partial sequence of the Indian isolate from Gorakhpur (accession no. MZ353593.1). The sequence PP952075 displayed 100% identity indicating no amino acid change, whereas the sequence PP952079 displayed 81.37% identity.

### 3.5 Seasonal trends in ST positivity in humans, animal reservoirs and mites

Among the 916 AUFI samples collected over 19 months, the ST positivity rate was highest in April (20%) and May (27.27%) (Figure 1). Among rodents/shrews trapped over a 12-month period, the highest positivity rate (33.33%) was observed in January and October. For mite pools, the highest positivity rate was recorded in June (7.25%).

## 4 Discussion

Scrub typhus is an endemic but frequently underdiagnosed zoonotic rickettsial disease, accounting for a significant proportion of acute undifferentiated febrile illness (AUFI) cases across multiple states in India. Outbreaks of scrub typhus are being reported over the past two decades in all the parts of the country, covering all the diverse climatic zones. As a vector-borne zoonosis, its burden is determined by the complex interactions between vector mites, animal reservoirs, and humans, the accidental hosts. Therefore, the present study aimed to investigate the genetic heterogeneity of *O. tsutsugamushi* strains across the human–animal–vector interface.

In the current study, of the 916 AUFI samples collected over 19 months from villages in and around Puducherry, ST infection was identified in 40 cases. The disease was more frequently seen in middle-aged men, likely due to occupational exposure to mite-infested environments, similar to the pattern reported in the earlier studies conducted in Kerala and Tamil Nadu (Varghese et al., 2013; Jyothi et al., 2015). The observed prevalence rate of 4.37% is considerably lower than the national prevalence of 25.3% reported in a systematic review (Devasagayam et al., 2021). The discordance observed could largely be attributed to the differences in diagnostic approaches. While the majority of studies relied on serological diagnostic tools especially IgM and IgG ELISA, we employed molecular diagnostics that enable the genetic characterization of the pathogen. PCRs are highly sensitive only during the window period of bacteremia. With the onset of adaptive immune response, the immunoglobulin levels rise, and the pathogenic load often decreases to a level undetectable by PCR. Hence, the samples collected quite later in the course of infection might have been undiagnosed by PCR, leading to the observed lower prevalence rate. In addition, the guidelines on the management of AUFI in adults, published by ICMR in 2019, insisted on the screening of patients with fever more than 5 days for ST and an optional empirical treatment with doxycycline in patients turning out to be negative in the rapid tests for dengue and malaria (ICMR guidelines on “Treatment guidelines for Antimicrobial use in common syndromes, 2019”). This in turn, could have cleared the pathogen load, hence rendering the PCR assay negative before sampling. More importantly, the study employed Real-time PCR targeting the 47 kDa gene and the Nested PCR targeting the 56 kDa gene, which were adopted from standard references. These assays have been reported to exhibit sensitivities of 75 ± 32% (range: 67.8–81.8%) and 97 ± 47% (range: 93.8–99.3%), respectively, with specificities of 100% (95.4–100%) and 100% (96.5–100%) (Kannan et al., 2020). This inherent variability in assay sensitivity, as reported in the literature, justifies the difference in the detection rates observed between the two tests in our study. This diagnostic limitation should also have contributed to the observed low prevalence estimates. Further, other factors such as the sampling bias due to case enrollment being limited to only three primary health care centers and variations in the sample collection, preservation, and transport might also have affected the positivity rate.

Rodents and shrews play a crucial role in the enzootic maintenance of *O. tsutsugamushi*, as evidenced by multiple studies (Devaraju et al., 2020; Balasubramanian et al., 2024), including the current investigation. Studies in animal models indicated that the bacteria remain detectable by PCR in multiple organs up to 84 days post-infection (Soong et al., 2016), and the viable rickettsiae have been found in rodent kidneys up to 4 months (Strickman et al., 1994). Our previous study reported a prevalence of 14.81% in rodents/shrews trapped even from areas without any reports of human cases of ST highlighting the risk of outbreak (Devaraju et al., 2020). The observed prevalence of 11.52% in this longitudinal study also reiterates the role of rodents and shrews in the enzootic maintenance of the pathogen and positive human infection confirms the transmission.

Sharma et al., reported a chigger index of 0.69 per rodent as a risk factor for ST outbreaks (Sharma, 2013). During the outbreak of ST in Himachal Pradesh (Kumar et al., 2004) and Gorakhpur (Sadanandane et al., 2021), chigger index of 2.46 and 5.3 was reported, respectively. In Puducherry, higher chigger indexes (41.1, 12.97, and 10.28 per animal) have been reported consistently in the recent past (Candasamy et al., 2016; Devaraju et al., 2020; Ritu et al., 2024). Following the same trend, in the current study a higher chigger index of 16.23 per animal was recorded. Month wise chigger index ranged from 1.95 (February) to 32.67 (June) per animal (Figure 1). Such a higher chigger index indicates a greater risk for ST outbreak during favorable climatic conditions. However, among the 296 pools of mites screened for ST, only 7 pools (2.36%) from 5 shrews (3 ST^+^ and 2 ST^−^) were tested positive for ST. A similar trend of low mite positivity was observed in our previous studies (Devaraju et al., 2020; Eikenbary et al., 2024) and the possible explanations include: (i) the potential DNA degradation during sample processing or storage, (ii) limitations inherent to the pooling strategy such as dilution of low-copy targets to the levels below the threshold of detection in PCR assays, (iii) the possibility of genuine low infection prevalence in the sampled mite population and (iv) other biological factors such as the host specificity and variability in vector competence. To address such issues, statistical modeling to determine the optimal pool size that balances sensitivity and cost-effectiveness, development of improved extraction protocols and internal controls to monitor DNA quality and standardization of operating protocols for the molecular xenomonitoring of mites for *Orientia* infection is suggested.

Globally, more than 40 strains of *O. tsutsugamushi* have been reported including the prototype strains like Kato, Karp and Gilliam (Nallan et al., 2025). Multiple serotypes of *O. tsutsugamushi* have been reported from various regions in India, showing significant geographic diversity. The most commonly identified strains in humans include the Karp-like and Kato-like genotypes (Varghese et al., 2013, 2015). In 2017, findings from Puducherry and its surrounding border areas in Tamil Nadu revealed the presence of Karp and Gilliam prototypes along with other serotypes such as Kuroki, Boryong, and Kato in humans (Anitha et al., 2017). Following the same trend, Karp-like and Gilliam-like strains were identified as the predominant circulating serotypes in Puducherry in the current study as well. Karp strain has been associated with longer stay in the hospital, with the involvement of multiple organs compared to the Gilliam strain. Further, the molecular evolutionary rate identified by Bayesian time scale analysis aligns with that reported earlier for *O. tsutsugamushi* with the TMRCA value suggesting the long-term circulation and diversification of the pathogen (Wongprompitak et al., 2015). Thus, the study highlights the co-circulation of strains with varying degree of virulence in Puducherry and the need to establish serotype-specific clinical manifestations existing in the region.

In addition to the predominant Karp and Gilliam-like strains, the current study also reports the circulation of TA678-like strain of *O. tsutsugamushi* for the first time in Puducherry in a shrew and vector mites. Although considered a less common genotype compared to Karp, Kato, or Gilliam, TA678 has been sporadically reported across parts of Southeast Asia and India, indicating a broader geographical distribution than previously recognized. The strain has been earlier reported in rodents and mites from Gorakhpur, Uttar Pradesh, which falls in the northernmost part of the country, during ST outbreaks significantly associated with Acute Encephalitis Syndrome (AES) (Sadanandane et al., 2018, 2021). In contrast, the incidence of such AES in ST patients has been only sparsely reported in South India. The above observation suggests that this new serotype may have recently entered the region, and its impact on clinical severity and outcome have to be monitored. In addition, the presence of TA678 in vectors without corresponding human cases reflects, host-specific transmission barriers, or underdiagnosis due to limited strain-specific molecular surveillance. Continued molecular monitoring and correlation with clinical data in human cases are essential to understand its unique virulence characteristics and associated variations in the disease severity compared to the other prevalent strains.

The genetic heterogeneity among different strains of the bacterium *O. tsutsugamushi* influences its antigenicity, which in turn affects the immune response, contributing to variations in disease severity and clinical outcomes across endemic regions. This factor also affects the diagnostic efficacy of the tools, as the real-time PCR probes commonly used can detect only certain prototypes, but not all the strains (Tantibhedhyangkul et al., 2017). The Karp genotype, in particular, has been consistently linked to severe clinical manifestations such as acute respiratory distress syndrome, hepatitis, renal failure, and thrombocytopenia (Chunduru et al., 2023). The higher prevalence of Karp-like strains identified in the current study suggests an increased likelihood of the disease progressing to more severe clinical outcomes if not promptly diagnosed and treated. Accordingly, in our study we encountered fatality in two cases due to severe complications such as thrombocytopenia, acute kidney injury and myocarditis. Among them, PCR positivity for the 56 kDa gene was observed in only one, and was identified as Karp-like suggesting its potential link to severe clinical outcomes. This underscores the need for ongoing surveillance and preparedness as Karp-like strains circulate in the region.

**Figure 3:**
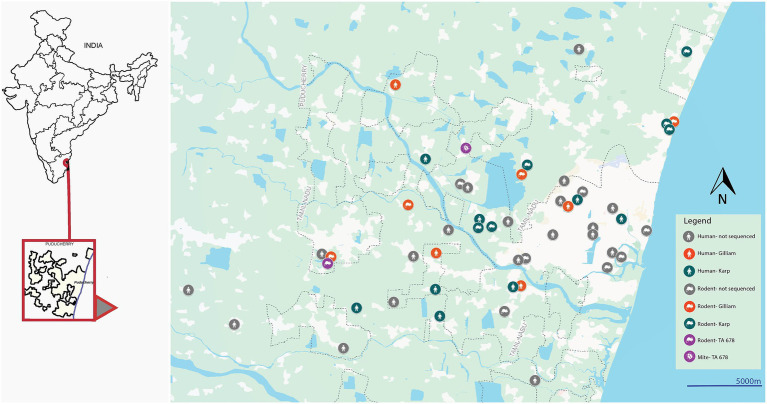
Map showing rodent/shrew, human, and chigger mite positive cases in and around Puducherry.

The pairwise genetic distance analysis revealed a clear pattern of genetic clustering based on serotype, with strains of the same serotype—Karp and Gilliam—exhibiting close genetic relationships (distance <0.1), and significantly higher divergence observed between different serotypes. Among the Karp-like strains, the lowest observed genetic distance was 0.05, indicating strong relatedness between human and rodent isolates, particularly for strains such as PP935509 and PP935510, suggesting active local transmission cycles. However, the human Karp strain PP935505 (Ulundurpet, Kallakurichi district) stood out as genetically distinct, showing no close association with any rodent or mite strains. Though the patient reported to a tertiary health care center in Puducherry, his residence was geographically distant from Puducherry. This supports the likelihood of an infection acquired outside the local transmission ecosystem, accounting for its divergence. Similarly, two TA678-like strains—one from a shrew (PP952075, Madagadipet) and one from a chigger mite (PP952079, Thuthipet)—were also genetically distinct and lacked close association with each other or with any other strain, suggesting either separate introductions or independent evolution in distinct ecological niches (Figure 3). Notably, within the Gilliam group, the human strain PP935511 showed a very high degree of similarity (distance = 0.03) with five Gilliam strains from shrews across different localities (Madagadipet, Poothurai, Periyababusamudram, and Bommayarpalayam). Another human Gilliam strain, PP935503 (Suthukeny, Villianur), also shared a close genetic relationship specifically with the Poothurai shrew strain PP952078. The observed genetic similarity strongly suggests sustained zoonotic transmission within this ecological niche.

The observed variability in amino acid sequences of the strains, when aligned with the full-length reference strains, suggests that the 56 kDa TSA gene can withstand a large amount of variation, as has been previously reported for the 47 kDa HtrA gene (Jiang et al., 2013). Notably, the human Karp strain PP935505, originating from a geographically distant location, exhibited the highest genetic distance and, as expected, the lowest amino acid identity. With respect to the TA678-like serotype, the chigger-derived sequence PP952079 exhibited the highest identity with the reference sequence (GU446611.1), which was also derived from chiggers. It was also observed that the shrew-derived sequence of TA678, PP952075 exhibited the highest identity with an Indian strain (MZ353593.1) detected in a shrew from Gorakhpur. In addition, a few cluster of strains were identical with the same translated amino acid sequences (i.e.), Gilliam: PP935508 and PP935518, Karp: PP952071, PP952074 and PP952076, Karp: PP935510, PP935512, PP935514 and PP935515 and Karp: PP935509 and PP935517 reaffirming the local transmission of the strains. Future studies should aim to obtain and analyze complete genomic data to enable comprehensive mutation analysis across additional genomic regions. Given the high mutation rates in rickettsiae which is attributable to their short generation times and large population sizes, such investigations are essential to better understand the genetic variability and evolutionary dynamics of the pathogen.

Scrub typhus exhibits a clear seasonal pattern influenced by environmental and climatic conditions. In India, the disease is most prevalent during the post-monsoon and cooler months (October to January), though regional variations exist (Mathai et al., 2003; Candasamy et al., 2016). These seasonal changes impact the abundance and activity of vectors and reservoirs, thereby influencing transmission dynamics. In the present study from Puducherry, while rodents/shrews showed the highest ST positivity during the cooler months (October and January) aligning with the above trend, chigger mite pools peaked in June. This indicated an increased vector activity during the early monsoon and a probable lag period between detectable positivity of the pathogen in mites and rodents/shrews. In the case of humans, though a noticeable increase in the cases was observed during the cooler months (November–January), the highest ST positivity occurred during the summer, particularly in April and May. As discussed earlier, this discrepancy in the month-wise positivity in human cases reflects the under-estimation of ST positives contributed by the choice of diagnostic tests used. The major thrust of the paper was to characterize the genetic heterogeneity of the pathogens and hence, human cases were detected only by molecular tests. PCR-based detection is known to be reliable only during the acute phase of infection, when antibody titres are low and pathogen levels are still detectable. Beyond the first week of clinical symptoms, PCR sensitivity declines significantly, making it unsuitable for late-phase diagnosis. Serological tests, such as ELISA, are therefore strongly recommended for detecting cases in AUFI patients presenting with fever for more than 5 days and to better understand the seasonal changes.

A major limitation of the study was the low infection rate detected in the screening of mites. The positivity rate did not correspond with the high chigger index observed in the field. This highlights the need for improved pooling strategies for xenomonitoring of ST in mites for future studies. Another important limitation was that only 29 of the 42 positives resulted in good-quality sequences suitable for genetic characterization. This might be because of the low pathogen load, resulting in less intense amplicon bands after the PCR. To circumvent this technical issue, we therefore, recommend the use of next-generation sequencing technologies, to generate reliable sequence data even from samples with low pathogen loads, mixed infection. Further, reliability only on PCR, absence of serological data, variability in diagnostic test accuracy, empirical treatment with doxycycline and minor differences in sample collection, transport, and storage across the three primary health centers may have introduced bias in the diagnostic outcomes and should be considered as limitations.

## 5 Conclusion

The current study revealed the higher prevalence of the Karp-like and Gilliam-like serotypes of *O. tsutsugamushi* among the animal reservoirs, vectors and the infected humans in and around Puducherry by phylogenetic analysis. With higher intra-serotype identity, abundance of infected animal hosts around human habitations, higher chigger infestation rates (39.78%) and higher chigger index (16.23%), this study highlights the ongoing transmission of the pathogen between animal reservoirs and humans through the mites. This emphasizes the need for regular zoonotic surveillance to predict disease outbreaks and to implement effective rodent control before the onset of congenial climatic conditions favoring the disease transmission. Further, with the first report of the TA678-like serotype among the reservoirs and vectors in the region, the study highlights the potential new introductions or strain evolution and reinforce the need for extensive molecular monitoring to track the newly emerging strains and to map their clinical significance.

## Data Availability

The datasets presented in this study can be found in online repositories. The names of the repository/repositories and accession number(s) can be found in the article/Supplementary material.

## Footnotes

^1^ https://www.ncbi.nlm.nih.gov/genbank/
