# Supplementary material for: A longitudinal molecular surveillance of genetic heterogeneity of Orientia tsutsugamushi in humans, reservoir animals, and vectors in Puducherry, India
Source: Front Microbiol. 2025 Aug 29;16:1634394. doi: 10.3389/fmicb.2025.1634394 (PMC12425938; doi:10.3389/fmicb.2025.1634394)
Supplement: Supplementary file 1 [file Data_Sheet_1.docx]

Supplementary Figure S1. Representative image of Molecular screening of DNA samples for the presence of *O. tsutsugamushi*. (A) Amplification Curve and (B) Heat Map of real time PCR, (C) Agarose gel electrophoresis indicating the amplification of 56kDa gene (483bp) by nested PCR.


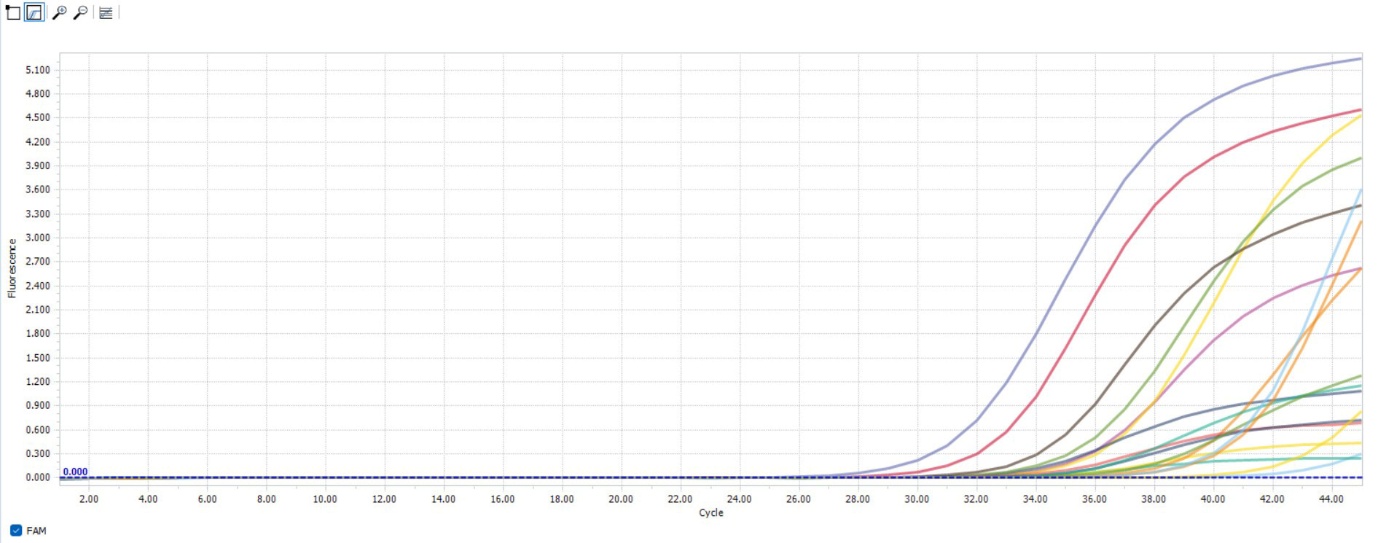


**A**


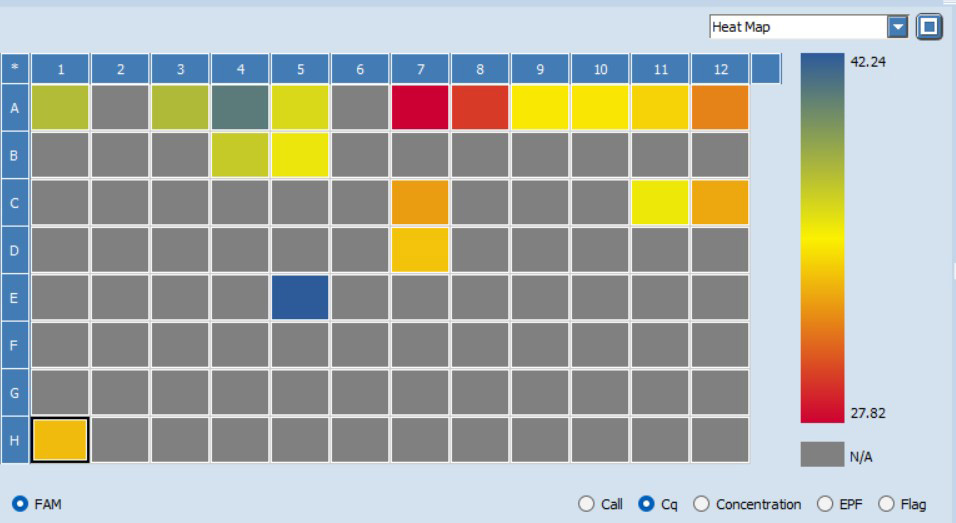


**B**


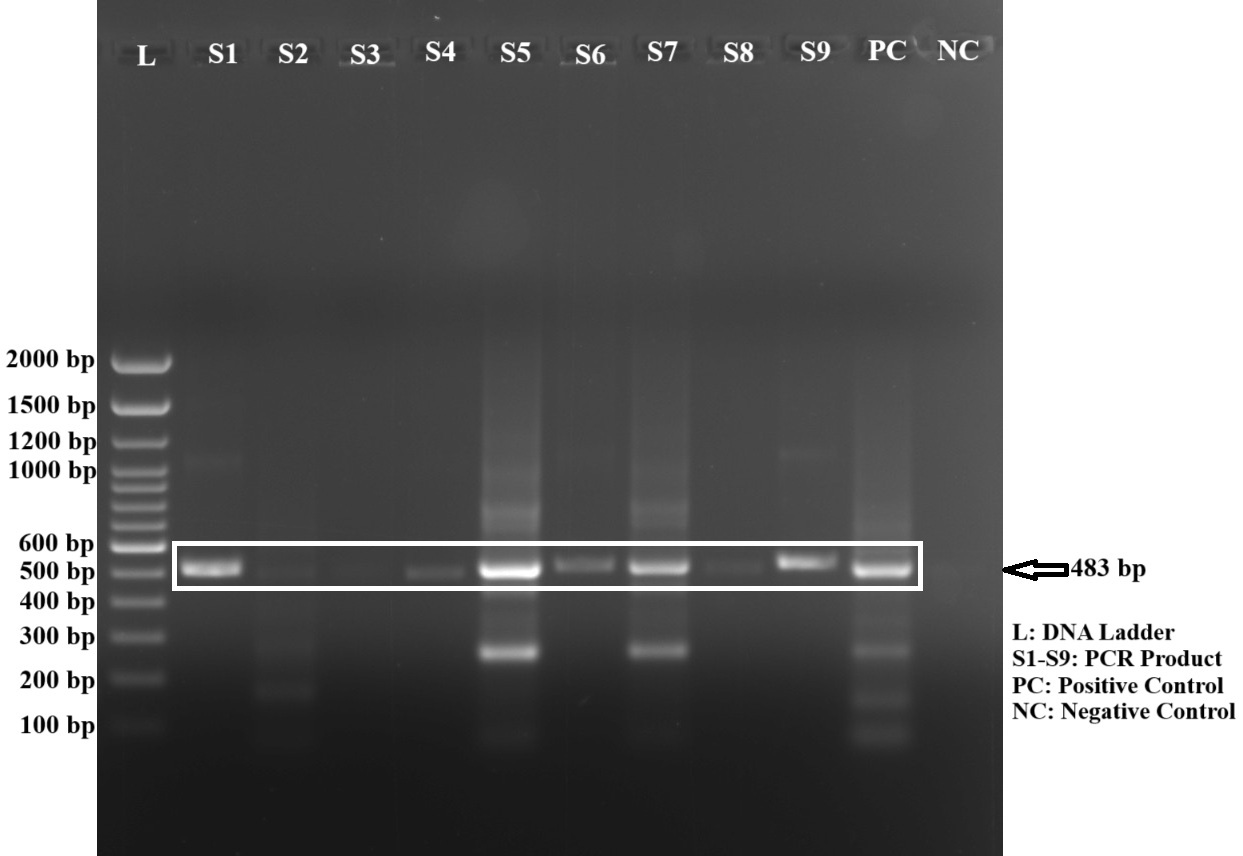


**C**
