# Supplementary material for: A longitudinal molecular surveillance of genetic heterogeneity of Orientia tsutsugamushi in humans, reservoir animals, and vectors in Puducherry, India
Source: Front Microbiol. 2025 Aug 29;16:1634394. doi: 10.3389/fmicb.2025.1634394 (PMC12425938; doi:10.3389/fmicb.2025.1634394)
Supplement: Supplementary file 2 [file Data_Sheet_2.docx]

Supplementary Table S1: Demographic details of Human samples positive for either Real Time PCR or nested PCR.

| Sl. No. | Sample Id | Location | Date of collection | Gender and Age | Days of Fever | Eschar Present/ Absent | Other organs | Other symptoms | Real Time PCR | Nested PCR |
| --- | --- | --- | --- | --- | --- | --- | --- | --- | --- | --- |
| 1 | L2211-1 | Oussudu, Villianur | 1/8/2022 | 45 Male | 7 | A | Thrombocytopenia (89k) | Cough with expectoration | Negative | Positive |
| 2 | L2211-45 | Suthukeny, Villianur | 30/11/2022 | 22 Female | 10 | A | Thrombocytopenia (83k), ESR 98, AST 486, ALT 337, ALP 151, Widal O- 1:320, l Lymphadenitis | Cough, breathlessness | Positive | Positive |
| 3 | L2211-46 | Villianur | 30/11/2022 | 68 Male | 7 | A | Right Lower lobe Pneumonia | Cough, loose stools | Positive | Negative |
| 4 | L2211-79 | Pudupakkam, Villupuram | 02/01/2023 | 50 Female | 7 | P | Hemoglobin 11.5, WBC count 13070, Platelet count 184k, ALP 413 | Headache, night sweats | Positive | Negative |
| 5 | L2211-86 | N/A | 13/10/2022 | N/A | N/A | N/A | N/A | N/A | Positive | Positive |
| 6 | L2211-99 | Pandacozhanallur, Bahour | 27/11/2022 | 67 Male | 8 | A | Thrombocytopenia (90k) | Myalgia, joint pains | Positive | Negative |
| 7 | L2211-102 | Arumparthapuram, Villianur | 30/11/2022 | 60 Male | 5 | A | AST 91, ALT-111  Thrombocytopenia (102K) | Chills, sweating | Positive | Negative |
| 8 | L2211-115 | Kandamangalam, Villupuram | 14/12/2022 | 44 Male | 1 | A | Thrombocytopenia (76K),  hepatosplenomegaly | Hiccups, Headaches | Positive | Negative |
| 9 | L2211-117 | Ulundurpet,  Kallakurichi | 15/12/2022 | 36 Male | 4 | A | Thrombocytopenia (81k) | Myalgia, headaches | Positive | Positive |
| 10 | L2211-128 | Vaniyampalayam, Villupuram | 24/12/2022 | 65 Female | 5 | P | Thrombocytopenia (66k) | Decreased appetite, metallic taste, breathlessness | Positive | Negative |
| 11 | L2211-145 | Ariyur, Villianur | 03/01/2023 | 45 Female | 7 | A | - | Body aches, vomiting | Positive | Negative |
| 12 | L2211-154 | Sapthagiri Nagar, Puducherry | 10/01/2023 | 62 Male | 4 | A | Thrombocytopenia (76k), | Cough. Breathlessness, constipation | Positive | Negative |
| 13 | L2211-159 | Madagadipet, Villianur | 14/01/2023 | 33 Male | 5 | A | Widal positive,  Plt-150k,  AST- 87, ALT- 112 | Myalgia, headache, neck pain, cough, deceased urine output | Positive | Negative |
| 14 | L2211-161 | M. Pudu palaiyam, Puducherry | 17/01/2023 | 63 Male | 7 | A | Thrombocytopenia (135K) | Constipation, vomiting | Positive | Positive |
| 15 | L2211-176 | Mettupalayam, Oulgaret | 27/01/2023 | 32 Male | 10 | A | Thrombocytopenia (147K), | Cough, | Positive | Negative |
| 16 | L2211-177 | Kizhur, Villianur | 27/01/2023 | 33 Male | 10 | A | Thrombocytopenia (122K), ALT 478, ALP- 304 | Cough | Positive | Negative |
| 17 | L2211-202 | Kendiyankuppam, Villupuram | 3/5/2023 | 40 Female | 7 | A | Thrombocytopenia (110k) | c&r cough | Positive | Negative |
| 18 | L2211-203 | Muthirapalayam, Oulgaret | 3/5/2023 | 24 Female | 7 | A | - | c&r cough | Positive | Negative |
| 19 | L2211-206 | Reddiyarpalayam, Oulgaret | 12/5/2023 | 37 Male | 10 | P | Thrombocytopenia (114k), WBC 15700, AST 300, ALT 301, hepatomegaly, myocarditis, AKI- Creatinine 1.45, Pulmonary edema | Vomiting, loose stools | Positive | Negative |
| 20 | L2211-289 | Shanmugapuram, Oulgaret | 16/6/2023 | 32 Male | 11 | A | - | Headache, cough, cold | Positive | Negative |
| 21 | L2211-290 | Saram, Oulgaret | 1/7/2023 | 36 Male | 3 | A | - | Body Aches | Positive | Negative |
| 22 | L2211-310 | Vazhudhavur, Villupuram | 20/7/2023 | 32 Female | 14 | P | Hepatosplenomegaly, Thrombocytopenia (51k) | Headache, abdominal pain, nausea, vomiting | Positive | Positive |
| 23 | L2211-346 | Poongunam, Villupuram | 02/04/2023 | 20 Male | 2 | A | Ventricular Tachycardia, | Palpitations, vomiting | Positive | Negative |
| 24 | L2211-353 | Poraiyur, Villianur | 12/04/2023 | 46 Male | 3 | A | AST- 127, ALP-168, Proteinuria, Thrombocytopenia- 121k, hepatomegaly, AKI- Creatinine 1.36 | Loose stools, neck pain | Positive | Negative |
| 25 | L2211-359 | Siruvanthadu, Villupuram | 30/04/2023 | 72 Male | 12 | A | Thrombocytopenia 50k, AKI- Creatinine 1.57, Myocarditis | Chest pain, vomiting, nausea, fainting | Positive | Negative |
| 26 | L2211-360 | Uruvaiyar, Villianur | 22/08/2023 | 29 Female | 7 | A | - | headache, cough, abdominal pain | Positive | Positive |
| 27 | L2211-521 | Melazhinjipattu, Cuddalore | 18/09/2023 | 58 Male | 14 | A | Proteinuria, Thrombocytopenia (40k), Transaminitis | hiccups | Positive | Negative |
| 28 | L2211-595 | Ramanathapuram, Villianur | 26/10/2023 | 38 Male | 10 | P | Leucopenia (2.82k) | Cough, Headache | Positive | Negative |
| 29 | L2211-718 | Koodapakkam, Villianur | 31/12/2023 | 46 Male | 7 | A | AKI- Creatinine 1.46, Platelets-153k | cough, breathlessness | Positive | Positive |
| 30 | L2211-753 | Uruvaiyar, Villianur | 26/12/2023 | 24 Female | 4 | A | Thrombocytopenia (100k), petechia seen, abdominal tenderness | Headache, nausea | Positive | Positive |
| 31 | L2211-834 | Siruvalai, Villupuram | 17/11/ 2023 | 75 Female | 3 | P | Pleural effusion, Thrombocytopenia (98k) | headache, cough, vomiting | Positive | Positive |
| 32 | L2211-836 | Koodapakkam, Villianur | 29/11/2023 | 40 Female | 14 | A | - | headache | Positive | Negative |
| 33 | L2211-861 | Nallathur, Cuddalore | 12/1/2024 | 18 Male | 10 | A | - | Nausea | Positive | Positive |
| 34 | L2211-868 | Pavandhur, Villupuram | 23/1/2024 | 47 Female | 4 | A | - | body aches | Positive | Positive |
| 35 | L2211-881 | Sivaranthagam, Villianur | 29/1/2024 | 44 Male | 10 | A | Thrombocytopenia (72k), Proteinuria, Glucosuria | cough, bodyache, bleeding gums | Positive | Positive |
| 36 | L2211-888 | Eripakkam, Bahour | 22/1/2024 | 50 Male | 7 | A | Thrombocytopenia (113k) | - | Positive | Positive |
| 37 | L2211-915 | Shanmugapuram, Oulgaret | 21/1/2024 | 59 Male | 8 | A | - | headache, vomiting | Positive | Positive |
| 38 | L2211-917 | Poraiyur, Villianur | 24/1/2024 | 21 Female | 7 | A | Thrombocytopenia (110k) | cough, cold | Positive | Negative |
| 39 | L2211-927 | Ariyur, Villianur | 22/1/2024 | 18 Female | 5 | A | Proteinuria | Cough, breathlessness | Positive | Positive |
| 40 | L2211-932 | Pakkiripalayam, Villupuram | 27/1/2024 | 57 Female | 13 | A | Leucopenia (3k), Thrombocytopenia (107k) | cough, headache | Positive | Positive |
