# Supplementary material for: A longitudinal molecular surveillance of genetic heterogeneity of Orientia tsutsugamushi in humans, reservoir animals, and vectors in Puducherry, India
Source: Front Microbiol. 2025 Aug 29;16:1634394. doi: 10.3389/fmicb.2025.1634394 (PMC12425938; doi:10.3389/fmicb.2025.1634394)
Supplement: Supplementary file 3 [file Data_Sheet_3.docx]

Supplementary Table S2: Month-wise data for number of traps set, number of Positive traps and trap positivity rate of the study area.

| Sl. No. | MONTHS | No. of traps set | No. of traps positive | Trap positivity rate (%) |
| --- | --- | --- | --- | --- |
| 1 | JANUARY | 60 | 9 | 15 |
| 2 | FEBRUARY | 140 | 20 | 14.29 |
| 3 | MARCH | 380 | 75 | 19.74 |
| 4 | APRIL | 300 | 64 | 21.33 |
| 5 | MAY | 220 | 62 | 28.18 |
| 6 | JUNE | 320 | 72 | 22.5 |
| 7 | JULY | 200 | 44 | 22 |
| 8 | AUGUST | 220 | 33 | 15 |
| 9 | SEPTEMBER | 260 | 57 | 21.92 |
| 10 | OCTOBER | 60 | 6 | 10 |
| 11 | NOVEMBER | 60 | 11 | 18.33 |
| 12 | DECEMBER | 60 | 7 | 11.66 |
