# Supplementary material for: A longitudinal molecular surveillance of genetic heterogeneity of Orientia tsutsugamushi in humans, reservoir animals, and vectors in Puducherry, India
Source: Front Microbiol. 2025 Aug 29;16:1634394. doi: 10.3389/fmicb.2025.1634394 (PMC12425938; doi:10.3389/fmicb.2025.1634394)
Supplement: Supplementary file 4 [file Data_Sheet_4.docx]

Supplementary Table S3: Area-wise data for number of traps set, number of Positive traps, trap positivity rate and number of species collected from the study area.

| Sl. No. | Area of Collection | No. of traps set | No. of traps Positive | Trap positivity rate (%) | *Suncus murinus* | *Rattus rattus* | *Bandicota indica* |
| --- | --- | --- | --- | --- | --- | --- | --- |
| 1 | Thuthipet, Villianur | 180 | 26 | 14.44 | 21 | 5 | 0 |
| 2 | Bahour | 120 | 33 | 27.5 | 26 | 7 | 0 |
| 3 | Bommayarpalayam, Puducherry | 280 | 52 | 18.57 | 45 | 6 | 1 |
| 4 | Boulevard, Puducherry | 20 | 15 | 75 | 1 | 3 | 11 |
| 5 | ICMR-VCRC Quarters, Puducherry | 20 | 1 | 5 | 1 | 0 | 0 |
| 6 | Kalapet, Oulgaret | 160 | 27 | 16.88 | 22 | 5 | 0 |
| 7 | Kanagachettikulam, Puducherry | 40 | 8 | 20 | 6 | 2 | 0 |
| 8 | Keerapalaiyam, Puducherry | 20 | 6 | 30 | 3 | 3 | 0 |
| 9 | Koodappakkam, Villianur | 120 | 37 | 30.83 | 34 | 3 | 0 |
| 10 | Koonichampet, Villianur | 60 | 17 | 28.33 | 16 | 1 | 0 |
| 11 | Korkadu, Bahour | 160 | 38 | 23.75 | 36 | 2 | 0 |
| 12 | Krishna Nagar, Puducherry | 20 | 2 | 10 | 0 | 2 | 0 |
| 13 | Madagadipet, Villianur | 100 | 17 | 17 | 14 | 3 | 0 |
| 14 | Mortandi, Villupuram | 20 | 1 | 5 | 1 | 0 | 0 |
| 15 | Murungampakkam, Puducherry | 20 | 2 | 10 | 0 | 2 | 0 |
| 16 | Ramanathapuram, Villianur | 20 | 1 | 5 | 1 | 0 | 0 |
| 17 | Poothurai, Villupuram | 200 | 30 | 15 | 26 | 3 | 1 |
| 18 | Navarkulam, Puducherry | 20 | 2 | 10 | 2 | 0 | 0 |
| 19 | Thavalakuppam, Puducherry | 160 | 38 | 23.75 | 29 | 8 | 1 |
| 20 | Poraiyur, Villianur | 20 | 1 | 5 | 1 | 0 | 0 |
| 21 | Periyababusamudram, Villupuram | 20 | 4 | 20 | 4 | 0 | 0 |
| 22 | Thengaithittu, Puducherry | 120 | 27 | 22.5 | 22 | 5 | 0 |
| 23 | Sedarapet, Villianur | 20 | 1 | 5 | 1 | 0 | 0 |
| 24 | Thirukkanur, Villianur | 80 | 16 | 20 | 15 | 1 | 0 |
| 25 | Villianur | 200 | 43 | 21.5 | 36 | 7 | 0 |
| 26 | Velrampet, Tallarevu | 20 | 7 | 35 | 6 | 0 | 1 |
| 27 | Vazhakulam, Tallarevu | 20 | 4 | 20 | 1 | 3 | 0 |
| 28 | White Town, Puducherry | 20 | 3 | 15 | 2 | 1 | 0 |
| 29 | Veterinary College, Oulgaret | 20 | 1 | 5 | 1 | 0 | 0 |
