# Supplementary material for: A longitudinal molecular surveillance of genetic heterogeneity of Orientia tsutsugamushi in humans, reservoir animals, and vectors in Puducherry, India
Source: Front Microbiol. 2025 Aug 29;16:1634394. doi: 10.3389/fmicb.2025.1634394 (PMC12425938; doi:10.3389/fmicb.2025.1634394)
Supplement: Supplementary file 5 [file Data_Sheet_5.docx]

Supplementary Table S4: Demographic details of rodent/shrew and mite’s samples positive for either Real time PCR or nested PCR. (“*” indicate two shrews were PCR positive only for chigger mites).

| Sl. No. | Sample ID | Area of Collection | Date of Collection | Rodent type | Sex | Mites P/A | Real time PCR positive sample | Nested PCR positive sample |
| --- | --- | --- | --- | --- | --- | --- | --- | --- |
| 1 | AR91 | Thuthipet, Villianur | 06/01/2022 | *Suncus murinus* | Female | Yes | - | Blood |
| 2 | AR95 | Korkadu, Bahour | 07/01/2022 | *Suncus murinus* | Female | No | Blood | Blood |
| 3 | AR97 | Korkadu, Bahour | 07/01/2022 | *Suncus murinus* | Female | Yes | - | Blood |
| 4 | AR99 | Thuthipet, Villianur | 15/02/2022 | *Rattus rattus* | Male | Yes | - | Lungs |
| 5 | AR100 | Thuthipet, Villianur | 15/02/2022 | *Suncus murinus* | Female | No | Lungs | Lungs |
| 6 | AR109 | Ramanathapuram, Villianur | 17/02/2022 | *Suncus murinus* | Male | No | Lungs | Lungs |
| 7 | AR110 | Poothurai,Villupuram | 18/02/2022 | *Suncus murinus* | Male | Yes | - | Lungs |
| 8 | AR116 | Murungapakkam, Puducherry | 25/02/2022 | *Rattus rattus* | Male | No | Lungs | - |
| 9 | AR118 | Bommayarpalayam, Puducherry | 01/03/2022 | *Suncus murinus* | Female | Yes | Heart, Lungs | Heart, Spleen |
| 10 | AR119 | Bommayarpalayam, Puducherry | 01/03/2022 | *Suncus murinus* | Male | Yes | Heart, Lungs | Heart, Spleen |
| 11 | AR120 | Bommayarpalayam, Puducherry | 01/03/2022 | *Suncus murinus* | Male | Yes | Lungs | - |
| 12 | AR121 | Bommayarpalayam, Puducherry | 01/03/2022 | *Suncus murinus* | Male | Yes | Blood, Lungs | - |
| 13 | AR126 | Poothurai, Villupuram | 03/03/2022 | *Suncus murinus* | Male | Yes | Liver | - |
| 14 | AR132 | Villianur | 04/03/2022 | *Rattus rattus* | Male | No | Blood | Blood |
| 15 | AR147 | Korkadu, Bahour | 11/03/2022 | *Suncus murinus* | Male | No | Liver | - |
| 16 | AR148 | Korkadu, Bahour | 11/03/2022 | *Suncus murinus(Dead)* | Male | No | Lungs | - |
| 17 | AR167 | Boulevard, Puducherry | 16/03/2022 | *Rattus rattus* | Male | No | Heart, Lungs | - |
| 18 | AR168 | ICMR-VCRC Quarters, Puducherry | 16/03/2022 | *Suncus murinus* | Female | Yes | Lungs | - |
| 19 | AR170 | Periyababusamudram, Villupuram | 18/03/2022 | *Suncus murinus* | Male | Yes | Blood | - |
| 20 | AR172 | Periyababusamudram, Villupuram | 18/03/2022 | *Suncus murinus* | Female | Yes | Liver | Liver |
| 21 | AR173 | Bommayarpalayam, Puducherry | 21/03/2022 | *Suncus murinus* | Female | Yes | Lungs | Lungs |
| 22 | AR182 | Thengaithittu, Puducherry | 23/03/2022 | *Suncus murinus* | Female | Yes | Intestine | - |
| 23 | AR185 | Thengaithittu, Puducherry | 23/03/2022 | *Suncus murinus* | Male | Yes | Lungs | - |
| 24 | AR186 | Bahour | 28/03/2022 | *Suncus murinus* | Female | Yes | Blood | - |
| 25 | AR189 | Bahour | 28/03/2022 | *Suncus murinus* | Female | Yes | Blood, Lungs | - |
| 26 | AR197 | Thuthipet, Villianur | 05/04/2022 | *Suncus murinus* | Female | Yes | Liver | - |
| 27 | AR200 | Korkadu, Bahour | 06/04/2022 | *Suncus murinus* | Male | Yes | Brain | - |
| 28 | AR204 | Poothurai, Villupuram | 07/04/2022 | *Suncus murinus* | Female | Yes | Heart | - |
| 29 | AR212 | Bommayarpalayam, Puducherry | 12/04/2022 | *Suncus murinus* | Male | Yes | Lungs | Kidney, Intestine |
| 30 | AR226 | Bommayarpalayam, Puducherry | 20/04/2022 | *Suncus murinus* | Male | No | Lungs | - |
| 31 | AR231 | Madagadipet, Villianur | 21/04/2022 | *Suncus murinus* | Female | Yes | Blood | - |
| 32 | AR233 | Madagadipet, Villianur | 21/04/2022 | *Suncus murinus* | Female | Yes | Brain | - |
| 33 | AR235 | Madagadipet, Villianur | 21/04/2022 | *Suncus murinus* | Male | Yes | Lungs | - |
| 34 | AR257 | Thuthipet, Villianur | 05/05/2022 | *Suncus murinus* | Male | Yes | Lungs, 1 Mite pool | - |
| 35 | AR259 | Thuthipet, Villianur | 05/05/2022 | *Suncus murinus* | Female | Yes | Lungs, 1 Mite pool | 1 Mite pool |
| 36 | AR260 | Korkadu, Bahour | 06/05/2022 | *Suncus murinus* | Male | Yes | Lungs | - |
| 37 | AR261 | Korkadu, Bahour | 06/05/2022 | *Suncus murinus* | Female | No | Lungs | - |
| 38 | AR330 | Villianur | 07/06/2022 | *Suncus murinus* | Male | Yes | Blood,  Lungs | - |
| 39 | AR334 | Villianur | 07/06/2022 | *Suncus murinus* | Female | Yes | Blood | - |
| 40* | AR336 | Bommayarpalayam, Puducherry | 08/06/2022 | *Suncus murinus* | Female | Yes | 3 Mite pools | - |
| 41 | AR343 | Koodapakkam, Villianur | 09/06/2022 | *Suncus murinus* | Male | Yes | 1 Mite pool | Blood |
| 42 | AR344 | Koodapakkam, Villianur | 09/06/2022 | *Suncus murinus* | Female | No | Blood | Intestine |
| 43* | AR354 | Kalapet, Oulgaret | 14/06/2022 | *Suncus murinus* | Female | Yes | 1 Mite pool | - |
| 44 | AR356 | Kalapet, Oulgaret | 14/06/2022 | *Suncus murinus* | Female | Yes | Blood, Kidney | - |
| 45 | AR358 | Kalapet, Oulgaret | 14/06/2022 | *Suncus murinus* | Male | No | Blood, Brain, Kidney | Heart |
| 46 | AR360 | Thengaithittu, Puducherry | 15/06/2022 | *Suncus murinus* | Male | No | Blood | - |
| 47 | AR365 | Madagadipet, Villianur | 16/06/2022 | *Suncus murinus* | Female | Yes | - | Blood |
| 48 | AR376 | Poothurai, Villupuram | 27/06/2022 | *Suncus murinus* | Female | Yes | Blood, Heart, Liver | Blood, Liver |
| 49 | AR377 | Poothurai, Villupuram | 27/06/2022 | *Suncus murinus* | Female | Yes | - | Blood |
| 50 | AR407 | Madagadipet, Villianur | 18/07/2022 | *Suncus murinus* | Male | Yes | - | Blood |
| 51 | AR506 | Thuthipet, Villianur | 21/09/2022 | *Suncus murinus* | Male | Yes | Blood | - |
| 52 | AR507 | Kalapet, Oulgaret | 22/09/2022 | *Suncus murinus* | Female | Yes | Blood | - |
| 53 | AR510 | Kalapet, Oulgaret | 22/09/2022 | *Suncus murinus* | Male | No | Blood | - |
| 54 | AR525 | Bommayarpalayam, Puducherry | 10/10/2022 | *Suncus murinus* | Male | No | Blood, Liver | - |
| 55 | AR526 | Bommayarpalayam, Puducherry | 10/10/2022 | *Suncus murinus* | Male | Yes | Blood, Liver | - |
