# Supplementary material for: A longitudinal molecular surveillance of genetic heterogeneity of Orientia tsutsugamushi in humans, reservoir animals, and vectors in Puducherry, India
Source: Front Microbiol. 2025 Aug 29;16:1634394. doi: 10.3389/fmicb.2025.1634394 (PMC12425938; doi:10.3389/fmicb.2025.1634394)
Supplement: Supplementary file 6 [file Data_Sheet_6.docx]

Supplementary Table S5. Distribution of the various serotypes of *Orientia tsutsugamushi* isolates among infected humans, rodents/shrews and chigger mites prevailing in and around Puducherry.

| Sl. No. | Geographical location | Source of isolate | GenBank accession number | Serotype |
| --- | --- | --- | --- | --- |
| 1 | Suthukeny, Villianur | Human | PP935503 | Gilliam like |
| 2 | N/A | Human | PP935504 | Gilliam like |
| 3 | Ulundurpet, Kallakurichi | Human | PP935505 | Karp like |
| 4 | M. Pudupalaiyam, Puducherry | Human | PP935506 | Karp like |
| 5 | Vazhudhavur, Villupuram | Human | PP935507 | Gilliam like |
| 6 | Uruvaiyur, Villianur | Human | PP935508 | Gilliam like |
| 7 | Koodapakkam, Villianur | Human | PP935509 | Karp like |
| 8 | Uruvaiyur, Villianur | Human | PP935510 | Karp like |
| 9 | Siruvalai, Villupuram | Human | PP935511 | Gilliam like |
| 10 | Nallathur, Cuddalore | Human | PP935512 | Karp like |
| 11 | Pavandhur, Villupuram | Human | PP935513 | Gilliam like |
| 12 | Sivaranthagam, Villianur | Human | PP935514 | Karp like |
| 13 | Eripakkam, Bahour | Human | PP935515 | Karp like |
| 14 | Shanmugapuram, Oulgaret | Human | PP935517 | Karp like |
| 15 | Ariyur, Villianur | Human | PP935518 | Gilliam like |
| 16 | Pakkiripalayam, Villupuram | Human | PP935519 | Karp like |
| 17 | Periyababusamudram, Villupuram | Shrew (*Suncus murinus*) | OR689573 | Gilliam like |
| 18 | Bommayarpalayam, Puducherry | Shrew (*Suncus murinus*) | OR689574 | Gilliam like |
| 19 | Bommayarpalayam, Puducherry | Shrew (*Suncus murinus*) | PQ037255 | Karp like |
| 20 | Bommayarpalayam, Puducherry | Shrew (*Suncus murinus*) | OR689572 | Karp like |
| 21 | Koodapakkam, Villianur | Shrew (*Suncus murinus*) | PP952071 | Karp like |
| 22 | Koodapakkam, Villianur | Shrew (*Suncus murinus*) | PP952077 | Karp like |
| 23 | Kalapet, Oulgaret | Shrew (*Suncus murinus*) | PP952076 | Karp like |
| 24 | Madagadipet, Villianur | Shrew (*Suncus murinus*) | PP952072 | Gilliam like |
| 25 | Poothurai, Villupuram | Shrew (*Suncus murinus*) | PP952073 | Gilliam like |
| 26 | Poothurai, Villupuram | Shrew (*Suncus murinus*) | PP952078 | Gilliam like |
| 27 | Poothurai, Villupuram | Shrew (*Suncus murinus*) | PP952074 | Karp like |
| 28 | Madagadipet, Villianur | Shrew (*Suncus murinus*) | PP952075 | TA678 like |
| 29 | Thuthipet, Villianur | Mite pool (AR259) | PP952079 | TA678 like |
