# Supplementary material for: A longitudinal molecular surveillance of genetic heterogeneity of Orientia tsutsugamushi in humans, reservoir animals, and vectors in Puducherry, India
Source: Front Microbiol. 2025 Aug 29;16:1634394. doi: 10.3389/fmicb.2025.1634394 (PMC12425938; doi:10.3389/fmicb.2025.1634394)

Supplementary Figure S2: Maximum clade credibility tree depicting the posterior probability of the study and reference sequences. The tree was generated using TreeAnnotator (BEAST) with a 95% highest posterior density (HPD) interval. Node labels indicate posterior probabilities, and branch lengths are scaled to time.


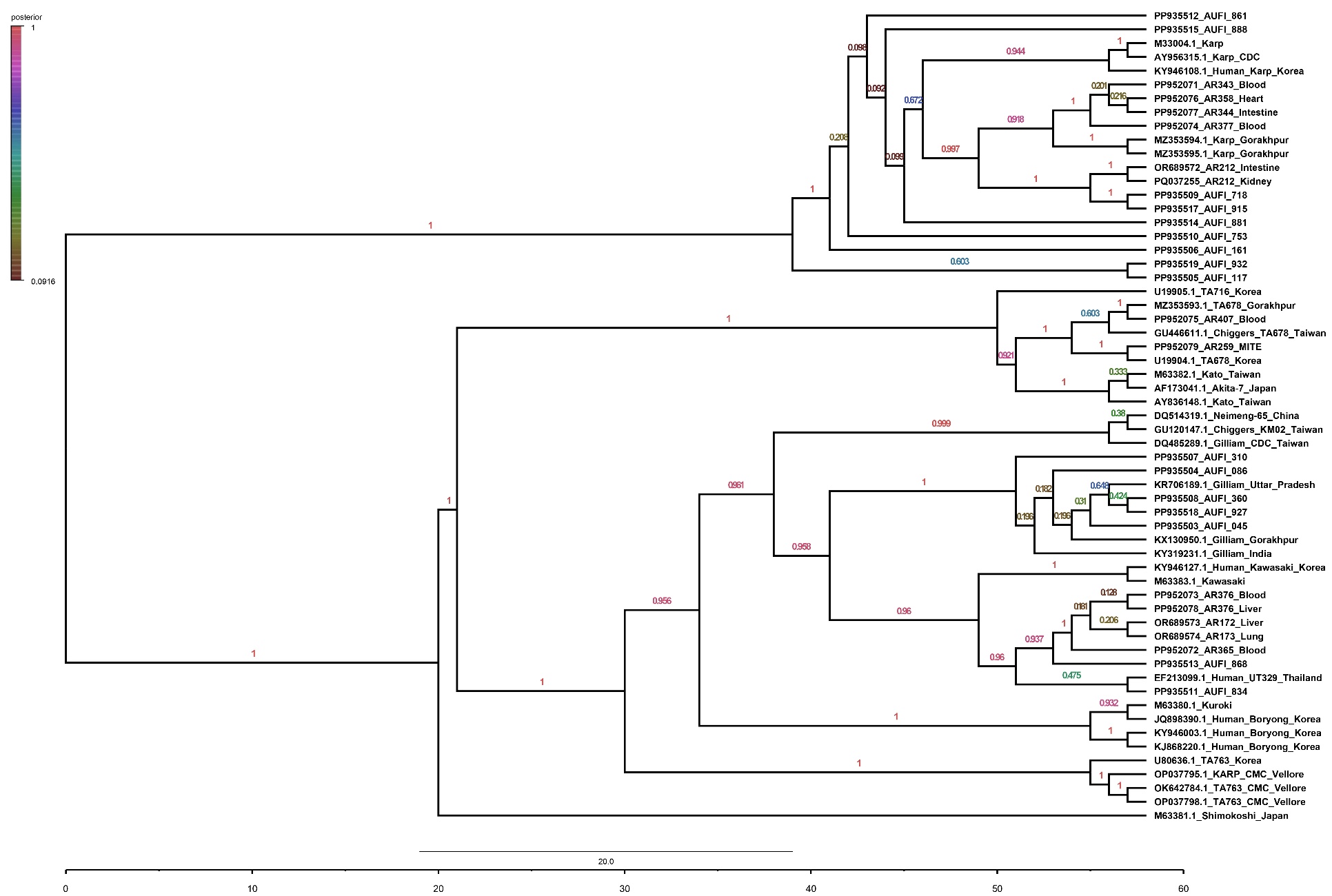

Supplement: Supplementary file 7 [file Data_Sheet_7.docx]
