# Supplementary material for: A longitudinal molecular surveillance of genetic heterogeneity of Orientia tsutsugamushi in humans, reservoir animals, and vectors in Puducherry, India
Source: Front Microbiol. 2025 Aug 29;16:1634394. doi: 10.3389/fmicb.2025.1634394 (PMC12425938; doi:10.3389/fmicb.2025.1634394)
Supplement: Supplementary file 8 [file Data_Sheet_8.docx]

Supplementary Table S6: Table representing the amino acid changes for the study sequences with reference to the full-length standard sequence of Gilliam, Karp and TA678.

GILLIAM

Reference: DQ485289.1

| Accession No. | Amino acid changes |
| --- | --- |
| PP952078  (AR376 Liver) | N198Q, N223D, L224F, Q231E, T251A, K268R, P269Q, D272K, G275N, D277E, T281A, G282P, E290G, Q291I, I292P |
| OR689573  (AR172 Liver) | L134I, T135K, P136L, R167Q, D176E, A179T, N198Q, N223D, L224F, Q231E, T251A, K268R, P269Q, D272K, G275N, D277E, T281A, G282P, E290G, Q291I, I292P, Q293R |
| PP952073  (AR376 Blood) | R167Q, D176E, A179T, N198Q, N223D, L224F, Q231E, T251A, K268R, P269Q, D272K, G275N, D277E, T281A, G282P, E290G, Q291I, I292P |
| PP952072  (AR365 Blood) | R167Q, D176E, A179T, N198Q, N223D, L224F, Q231E, T251A, K268R, P269Q, D272K, G275N, D277E, T281A, G282P, E290G, Q291I |
| OR689574  (AR173 Lung) | L134I, T135K, P136L, R167Q, D176E, A179T, N198Q, N223D, L224F, Q231E, T251A, K268R, P269Q, D272K, G275N, I276M, D277K, T281A, G282P, E290G, Q291I, I292P |
| PP935511  (AUFI 834) | R167Q, D176E, N198H, N223D, D225N, K268R, P269Q, D272K, G275N, D277E, T281A, G282P, E290G |
| PP935513  (AUFI 868) | Q160L, A161S, A163F, R167Q, D176E, N198H, D225N, K268R, P269Q, D272K, G275N, D277E, T281A, G282P, E290G, Q291I, I292P |
| PP935503  (AUFI 045) | R167-(deletion), L168-(deletion), V170I, E171K, Q172R, R173N, A174T, D176S, R177-(deletion), I178-(deletion), A179-(deletion), W180-(deletion), L181-(deletion), I202V, D225N, K262Q, S265R, D277N, G282A, E290G, Q291I, I292P |
| PP935504  (AUFI 086) | R167Q, I202V, D225N, K262Q, S265R, D277N, G282A, E290G, Q291I, I292P |
| PP935507  (AUFI 310) | T135K, P136L, R167Q, I202V, D225N, K262Q, S265R, D277N, G282A |
| PP935508  (AUFI 360) | D150C, R167Q, I202V, D225N, K262Q, S265R, D277N, G282A, E290G, Q291I, I292P, Q293R |
| PP935518  (AUFI 927) | D150C, R167Q, I202V, D225N, K262Q, S265R, D277N, G282A, E290G, Q291I, I292P, Q293R |

Note: X000Y indicates an amino acid change, where X is the reference amino acid, 000 is the position with respect to reference sequence, and Y is the amino acid in the study sequence.

KARP

Reference: AY956315.1

| Accession No. | Amino acid changes |
| --- | --- |
| PP952071  (AR343 Blood) | N212D, N219A, P220L, N223Q, P230R, G233D, A235E, E240D, H244L, T271I |
| PP952074  (AR377 Blood) | N212D, N219A, P220L, N223Q, P230R, G233D, A235E, E240D, H244L, T271I |
| PP952076  (AR358 Heart) | N212D, N219A, P220L, N223Q, P230R, G233D, A235E, E240D, H244L, T271I |
| PP952077  (AR344 Intestine) | Q166N, D171N, N212D, N219A, P220L, N223Q, P230R, G233D, A235E, E240D, H244L, T271I |
| OR689572  (AR212 Intestine) | P155R, Q159-(deletion), Q160-(deletion), Q166N, P167-(deletion), Q168-(deletion), D171N, N194D, N196H, D197N, N219I, P224-(deletion), P225-(deletion), N229Q, P300Q, N238D, H244Y, V247T, P279L, T291A, S292G, E300G, Q301I, I302P, Q303R |
| PQ037255  (AR212 Kidney) | L133I, T134K, P135L, P155R, Q159-(deletion), Q160-(deletion), A165G, Q166N, P167-(deletion), Q168-(deletion), D171N, N194D, D197N, N219I, P224-(deletion), P225-(deletion), N229Q, P230Q, N238D, H244Y, V247T |
| PP935514  (AUFI 881) | T271S, S292G, E300G |
| PP935515  (AUFI 888) | T271S, S292G, E300G |
| PP935512  (AUFI 861) | T271S, S292G, E300G |
| PP935510  (AUFI 753) | T271S, S292G, E300G |
| PP935506  (AUFI 161) | T271S, S292G, E300G, Q301I, I302P |
| PP935509  (AUFI 718) | P155C, Q160A, Q166N, Q168A, N194D, D197N, V221A, N229Q, P230Q, G233N, E240D, Q241H, E300G |
| PP935517  (AUFI 915) | P155C, Q160A, Q166N, Q168A, N194D, D197N, V221A, N229Q, P230Q, G233N, E240D, Q241H, E300G |
| PP935505  (AUFI 117) | D149G, F150I, G151A, I152L, D153S, P155C, N156L, P158K, Q159-(deletion), Q160-(deletion), Q161L, A162P, A164P, A165T, Q166F, P167M, Q168P, L169N, N170S, D171A, E172S, Q173V, R174G, A175I, A176P, A177S, R178G, W181L, L182A, N184C, C185L, A186I, G187N, I188L, D189P, Y190Q, R191P, V192T, K193T, N194M, D197N, P198A, N199A, -201G(insertion), -202I(insertion), M204R, V205G, N207A, P208L, I209A, L210I, L211R, N212L, P214K, Q215L, G216P, N217Q, N219T, P220I, V221M, G222P, P224-(deletion), P225-(deletion), Q226-(deletion), R227S, N229S, P230V, P231G, A232I, G233P, T271S, S292G, E300G, Q301I, I302P |
| PP935519  (AUFI 932) | Q166F, P167L, Q168P, N199A, T271S, S292G, E300G, Q301I, I302P, Q303R |

Note: X000Y indicates an amino acid change, where X is the reference amino acid, 000 is the position with respect to reference sequence, and Y is the amino acid in the study sequence.

TA678

Reference: GU446611.1

| Accession No. | Amino acid changes |
| --- | --- |
| PP952075  (AR407 Blood) | N142D, D154E, Q157R, V162N, V165A, A174T, H189N, A194V, A201D, R204Q, S212A, Q215P, V217A, G218D, Q219R, A221E, D222N, C223F, C233Y, I234V, T236A, M266V, P268Q, V272I, I275V, I288V, E289G |
| PP952079  (AR259 Mite) | L185Q, H189N, A194R, R204Q, N206D, N208H, P210L, S212G, G213Q, V217A, G218H, A221D, C223F, C233Y, I234V, T236V, V260A, R264S, M266V |

Note: X000Y indicates an amino acid change, where X is the reference amino acid, 000 is the position with respect to reference sequence, and Y is the amino acid in the study sequence.
